# Energy Dissipation Pathways in Few-Layer MoS_2_ Nanoelectromechanical Systems

**DOI:** 10.1038/s41598-017-05730-1

**Published:** 2017-07-18

**Authors:** Bernard R. Matis, Brian H. Houston, Jeffrey W. Baldwin

**Affiliations:** Naval Research Laboratory, Code 7130, Washington, DC 20375 United States

## Abstract

Free standing, atomically thin transition metal dichalcogenides are a new class of ultralightweight nanoelectromechanical systems with potentially game-changing electro- and opto-mechanical properties, however, the energy dissipation pathways that fundamentally limit the performance of these systems is still poorly understood. Here, we identify the dominant energy dissipation pathways in few-layer MoS_2_ nanoelectromechanical systems. The low temperature quality factors and resonant frequencies are shown to significantly decrease upon heating to 293 K, and we find the temperature dependence of the energy dissipation can be explained when accounting for both intrinsic and extrinsic damping sources. A transition in the dominant dissipation pathways occurs at *T* ~ 110 K with relatively larger contributions from phonon-phonon and electrostatic interactions for *T* > 110 K and larger contributions from clamping losses for *T* < 110 K. We further demonstrate a room temperature thermomechanical-noise-limited force sensitivity of ~8 fN/Hz^1/2^ that, despite multiple dissipation pathways, remains effectively constant over the course of more than four years. Our results provide insight into the mechanisms limiting the performance of nanoelectromechanical systems derived from few-layer materials, which is vital to the development of next-generation force and mass sensors.

## Introduction

Nanoelectromechanical systems (NEMS) derived from stable, atomically thin and ultralightweight two-dimensional (2D) materials like graphene^1–3^, the transition metal dichalcogenides (TMDs)^4, 5^, and phosphorene^6^ offer the prospect of coupling the mechanical degree of freedom to the unique properties of each material^7^ thus creating a new class of mechanically active architectures with novel electro- and opto-mechanical capabilities. Mechanical energy dissipation is ubiquitous in all NEMS and sets a fundamental limit on the performance of these systems. Measurements of the temperature, *T*, dependent energy dissipation, *Q* ^−1^ (where *Q* is the resonator quality factor), in graphene and monolayer WSe_2_ NEMS show a substantial increase in *Q* ^−1^ (over several decades) from liquid helium temperatures up to room temperature^1, 5^. Though *Q* has been shown to exceed 1.0 × 10^4^ in these systems at cryogenic temperatures (~1.0 × 10^5^ for graphene), the increase in mechanical energy dissipation with increasing *T* substantially reduces the *Q* values, which limits the room temperature force sensitivity of each resonator (since the limit of force sensitivity^2^ $dF\propto {(T/Q\omega )}^{1/2}$, where *ω* = 2π*f* and *f* is the resonator frequency).

In this work, we use an all-optical setup to characterize the *T* dependence of *Q* ^−1^ for few-layer MoS_2_ NEMS, which allows us to determine the dissipation mechanisms responsible for the experimentally observed *Q* values from *T* = 4.4 K up to room temperature. A pulsed blue diode laser (*λ* = 412 nm) modulated at frequency, *f*, thermoelastically excites the resonator into resonance while the reflected light intensity of a red *(λ* = 633 nm) helium-neon laser is monitored to detect the resonator motion. In all instances, care is taken to avoid absorptive heating and dynamical photothermal back-action from the lasers, by limiting the amount of laser power delivered to the sample^5^. All measurements are carried out in vacuum (pressure ~10^−6^ Torr), minimizing energy dissipation due to gas friction (see the Supplementary Information). The sample is mounted in front of an optical window within a variable temperature helium cryostat.

Room temperature measurements demonstrate fundamental and higher order modes in the 10–100 MHz range with measured *Q* on the order of ~100. Upon cooling to 4.4 K, *Q* reaches values on the order of 1.0 × 10^4^ while the fundamental mode increases in frequency by ~10 MHz. At 4.4 K, the experimentally determined Young’s Modulus, *E*, of the few-layer MoS_2_ resonator is ~178 GPa, which is within experimental error of the values obtained for single- and bi-layers, *E* ~ 270 ± 100 GPa and *E* ~ 200 ± 60 GPa, respectively^8^. We find that it is necessary to account for both intrinsic (phonon-phonon interactions) and extrinsic (clamping and electrostatic coupling to the substrate) sources of energy dissipation in order to explain the observed temperature dependence of *Q* ^−1^ while thermoelastic dissipation and dissipation due to surface losses do not play a significant role. The experimentally determined functional form of *Q* ^−1^(*T*) indicates a transition in the dominant energy dissipation pathways at *T* ~ 110 K with relatively larger contributions to *Q* ^−1^(*T*) from phonon-phonon and electrostatic interactions in the high temperature regime and larger contributions from clamping losses in the low temperature regime. Despite the presence of multiple dissipation pathways, measurements of the room temperature product *Qω* result in a force sensitivity of ~8 fN/Hz^1/2^ that is found to remain constant over the course of over four years, suggesting long-term durability and functionality of these few layer TMD NEMS.

## Results

### Optical characterization

**Figure 1:**
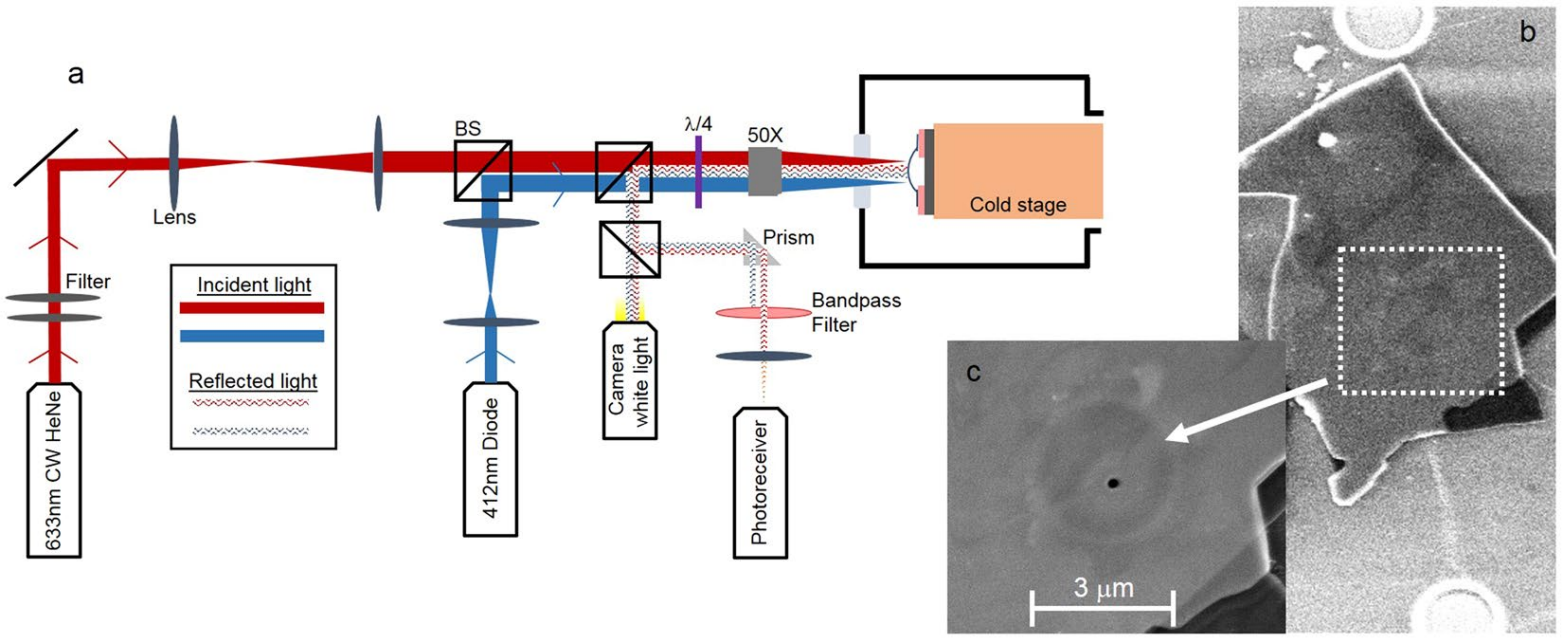
Optical interferometer and MoS_2_ resonator. (**a**) Schematic showing the optical interferometer, and sample mount within the variable temperature cryostat. BS = Beam Splitter. *λ*/4 = quarter-wave plate. (**b** and **c**) Scanning electron microscopy images of one of the MoS_2_ resonators tested within this study. The observable hole near the center of the resonator in (**c**) was made by ion milling with a focused ion beam to test the impact of trapped air beneath the MoS_2_ on the overall mechanical properties of the resonator.

The optical interferometer used in our experiments is shown schematically in Fig. 1a. Scanning electron micrographs of one of the circular, drumhead resonators used in this study are shown in Fig.’ s1b,c. Few-layer MoS_2_ flakes were mechanically exfoliated from bulk MoS_2_ crystals onto a SiO_2_/(doped)Si substrate with predefined circular trenches etched out of the SiO_2_. Atomic force microscopy was used to determine flake thickness. A total of three resonators were tested in this study, all of which showed the same qualitative behavior, and we present data from one representative resonator.

**Figure 2:**
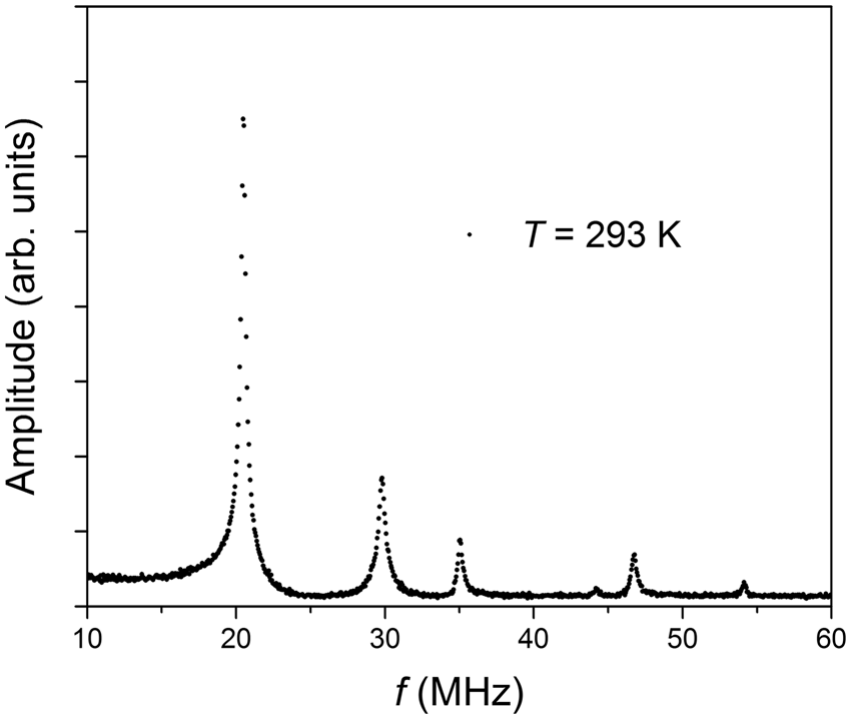
Room temperature mechanical resonance spectrum. Amplitude as a function of frequency, *f*, for the 17-nm-thick MoS_2_ resonator showing the fundamental mode (*f* ~ 21.8 MHz) and higher-order modes.

Figure 2 shows the measured mechanical amplitude versus frequency at room temperature for a 17-nm-thick (~26 monolayers) MoS_2_ resonator with a radius *r* ~ 3 μm. The choice to study few-layer resonators was for several reasons: particularly, for comparing energy dissipation pathways in the transition from bulk to single-atomic layer material, and to determine whether a few-layer MoS_2_ resonator could in fact possess the same exceptional mechanical properties as those of its single-layer counterparts (e.g. high *Q*) while simultaneously combining long-term durability against energy dissipation due to surfaces losses (e.g. from physisorbed species). For the data shown in Fig. 2, the fundamental mode is observed at *f* ~ 21.8 MHz, which shows the largest mechanical amplitude as well as higher-order vibrational modes. For the remainder of our temperature-dependent data analysis we focus our discussion on the fundamental mode. Determination of the resonant frequency and full-width-at-half-maximum, *Γ*, which is used in the determination of the quality factor *Q* = *f/Γ*, is done through a Lorentzian fitting of the square of the measured amplitude versus frequency.

**Figure 3:**
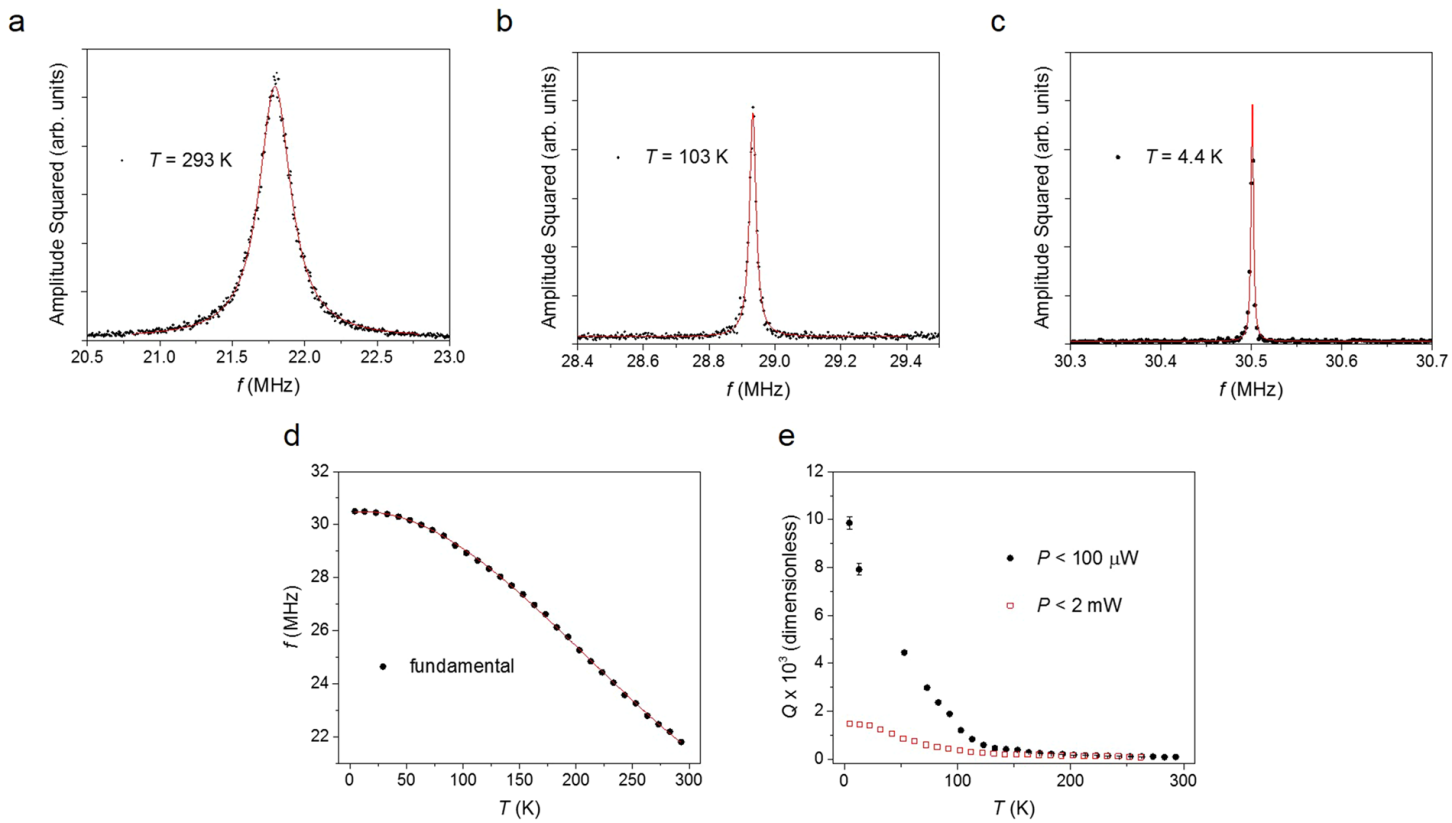
Temperature dependence of the MoS_2_ resonator fundamental mode. (**a–c**) Square of the amplitude as a function of frequency, *f*, for varying temperature, *T*. The solid red lines are Lorentzian fits to the data. (**d**) Fundamental frequency as a function of *T*. The solid red line is a fit to a third-order polynomial, which is used in the subsequent data analysis for determining energy dissipation pathways. (**e**) Resonator quality factor, *Q*, as a function of *T* for varying laser power, *P*.

Figures 3a–c show the amplitude squared versus frequency for three separate temperatures (293 K, 103 K and 4.2 K), which include the Lorentzian fits used to extract *f* and *Γ*. Lorentzian fits to the data shown in Fig.’ s3a–c yield *f* ~ 21.8 MHz and *Q* ~ 82 ± 1 at *T* = 293 K, and *f* ~ 31 MHz and *Q* ~ 9,857 ± 271 at *T* = 4.4 K. The increase in *Q* and *f* with decreasing *T* is consistent with prior studies on graphene and monolayer MoS_2_ and WSe_2_ NEMS^1, 5^. The strong temperature dependence of *f* has been associated with the thermal expansion of the resonator. Figure 3d shows the temperature dependence of the fundamental frequency, determined through Lorentzian fittings like those shown in Fig.’ s3a–c, along with a fit to a third order polynomial (see the Supplementary Information). The third order polynomial is used in the subsequent data analysis (see below) to accurately fit the temperature dependence of the experimentally observed energy dissipation (which can depend on *f*). Furthermore, the frequency modes for a circular plate resonator (in the limit of zero tension) can be expressed as^9^

$${f}_{mn}=(\pi t/4{r}^{2})\sqrt{E/3\rho (1-{s}^{2})}{({\beta }_{mn})}^{2}$$

where *t* ~ 17 nm is the plate thickness, *E* is the Young’s modulus, *ρ* ~ 5,060 kg/m^3^ is the resonator mass density^10^, *s* = 0.27 is the Poisson’s ratio for bulk MoS_2_^11^, and *β*_mn_ is the *n* ^th^ root of the *m* ^th^-order Bessel function (here, we use *β*_01_ = 2.4048 when considering the fundamental mode). At *T* = 4.4 K and with *f* ~ 31 MHz we find *E* ~ 178 GPa, which is in reasonable agreement with stretching and breaking results of few layer MoS_2_ films^8^.

Throughout our experiments care was taken to avoid absorptive heating and dynamical photothermal back-action, as well as to operate within the linear regime away from any non-linear Duffing behavior. Upon cooling to 4.4 K, maximal laser power, *P*, induced observable Duffing nonlinearities in the resonator amplitude versus *f* ^12, 13^. At 4.4 K, before capturing the data shown in Fig. 3, the laser power of each laser was reduced well below the minimum threshold required to observe Duffing behavior, toward the minimum power limit beyond which no resonance could be observed in the frequency spectrum, and to the point at which we perceived no observable changes in *f* and *Γ*. Figure 3e shows *Q* as a function of *T* for before and after this reduction in laser power. The power values given in the Fig. 3e legend represent upper limits, however laser power delivered to the sample is lower (see Methods). The data in Fig. 3e demonstrates that under minimal laser power conditions, *Q* values reaching 1.0 × 10^4^ can be achieved in these few-layer resonators at cryogenic temperatures.

### Dissipation mechanisms

**Figure 4:**
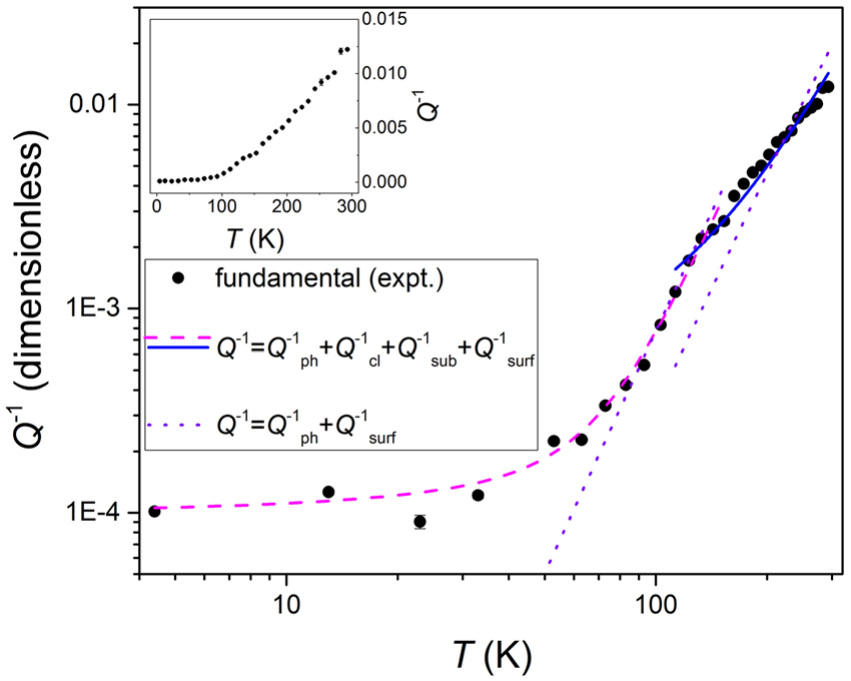
Temperature dependence of the energy dissipation. Inverse quality factor (energy dissipation), *Q* ^−1^, as a function of temperature, *T*. The solid black circles are the experimental data determined from measurements of the fundamental mode temperature dependence. The solid blue and dashed magenta lines are fits to the experimental data for *T* > 110 K and *T* < 110 K, respectively, which take into account four dissipation pathways: *Q* ^−1^_ph_ = phonon-phonon dissipation, *Q* ^−1^_cl_ = clamping dissipation, *Q* ^−1^_sub_ = dissipation from electrostatic interactions with the substrate, and *Q* ^−1^_surf_ = surface dissipation. The dotted violet lines are fits to the data within the high and low temperature ranges that only take into account *Q* ^−1^_ph_ and *Q* ^−1^_surf_. Inset: *Q* ^−1^ versus *T* on a linear scale. The *y*-axis units are dimensionless.

Measurements of *Q* ^−1^(*T*) allow us to identify the main causes of energy dissipation within the studied MoS_2_ NEMS, and the experimentally determined *Q* ^−1^(*T*) is shown in Fig. 4 (solid black circles). We identify two separate regimes for *Q* ^−1^(*T*) with a transition occurring at *T* ~ 110 K. Upon cooling, *Q* ^−1^(*T*) falls approximately an order of magnitude between 293 K and 110 K, with measured *Q* ^−1^(*T*) values of ~0.01 and ~1 × 10^−3^, respectively. For *T* < 110 K, we find that *Q* ^−1^(*T*) saturates to values of ~1.0 × 10^−4^ at the lowest temperatures, which results in quality factors as high as 10,000 at 4.4 K. The observed transition in the functional form of *Q* ^−1^(*T*) near *T* ~ 110 K suggests that different dissipation pathways govern the energy dissipation within the high and low temperature regimes.

We consider multiple energy dissipation mechanisms in an effort to explain the experimentally determined functional form of *Q* ^−1^(*T*). Dissipation due to phonon-phonon interactions, ${Q}_{ph}^{-1}(T)$, within the Akheiser regime^14, 15^ can be expressed as

$${Q}_{ph}^{-1}(T)=\frac{{C}_{v}{\gamma }^{2}T}{\rho {v}^{2}}\frac{\omega {\tau }_{ph}}{1+{\omega }^{2}{{\tau }_{ph}}^{2}}$$

where *γ* ~ 1.1 is Grüneisen’s constant^16^ for MoS_2_, *C*_*v*_ is the heat capacity per unit volume, *v* is the sound velocity within the MoS_2_ and *τ*_*ph*_ the phonon relaxation time. For multilayer MoS_2_^17^, *v* ~ 7.11 × 10^3^ m/s, which corresponds to *λ* ~ 326 μm at *f* ~ 21.8 MHz. The Akheiser regime is applicable in our samples since *λ* ≫ *L*_*ph*_ (where *L*_*ph*_ ~ 200 nm is the phonon mean free path^18^) and 1/*τ*_*ph*_ ≫ *ω* where ${\tau }_{ph}=3\kappa /{C}_{v}{v}^{2}$ ~ 2 ps with *κ* ~ 52 W/Km is the few-layer MoS_2_ thermal conductivity^19^ and the heat capacity is ref. 20 *C*_*v*_ ~ 1.89 × 10^6^ J/m^3^ K (see the Supporting Information for further discussion on the Akheiser regime). Within this regime, the oscillatory sound wavelength perturbs the phonon distribution from the equilibrium Planck form, while the restoration of the phonon gas to thermal equilibrium is accompanied by dissipation in the resonator. We further take into account clamping losses (energy radiated away from the resonator into the substrate), ${Q}_{cl}^{-1}$, which we take to be independent of temperature. Prior works have studied clamping losses in detail and have shown that such dissipation is temperature independent and arises purely from the geometry of the system^21, 22^.

We also take into account dissipation due to electrostatic interactions between the charges in the resonator and the doped silicon substrate, ${Q}_{sub}^{-1}(T)$. For the case of graphene, it has been shown theoretically that the room temperature dissipation is dominated by ohmic losses at the graphene and the metallic gate^21^. Here, the charge carriers within the resonator induce a time-dependent potential on the backgate, thus creating electron-hole pairs within the resonator and/or backgate. Electron-hole generation processes such as these results in energy dissipation, which can be the dominant energy dissipation pathway for 2D graphene-based NEMS. In this case, ${Q}_{sub}^{-1}(T)$ should increase linearly with temperature^21^. We note the importance of incorporating the linear in *T* term to the overall dissipation observed within our studied NEMS, as its inclusion is necessary for accurately describing the observed functional form of *Q* ^−1^(*T*). Unlike the case of monolayer graphene, however, we do not expect ${Q}_{sub}^{-1}(T)$ to be the dominant dissipation pathway within our few-layer resonators due to screening of the charge carriers within few-layer MoS_2_.

Room temperature measurements on multilayer MoS_2_ resonators have demonstrated a decrease in the quality factor with an increase in the surface to volume ratio^4^, which indicates a contribution from surface-related dissipation to the overall *Q* ^−1^(*T*). Therefore, we account for surface related dissipation using the known expression^23^

$${Q}_{surf}^{-1}(T)=\frac{{V}_{monolayer}}{V}\frac{E{\alpha }^{2}T}{{C}_{p}}\frac{\omega {\tau }_{s}}{1+{\omega }^{2}{{\tau }_{s}}^{2}}$$

where *E* ~ 178 GPa is the experimentally determined Young’s Modulus, *α* ~ 1 × 10^−6^ is the MoS_2_ average thermal expansion coefficient^24^, we assume *C*_*p*_ = *C*_*v*_ ~ 1.89 × 10^6^ J/m^3^ K, and ${\tau }_{s}={a}^{2}{C}_{p}/\kappa$ where *a* is the average crystallite size taken to be the diameter of the resonator (assuming surface-related effects across the entire resonator surface). The ratio ${V}_{monolayer}/V$ represents the ratio between the volumes of the damaged layer and the resonator as a whole and we assume the damaged layer to be the top MoS_2_ layer of the resonator, which is the layer most susceptible to physisorbed species.

In comparing these dissipation models to our data, we use the experimentally determined *f*(*T*) in determining *ω*(*T*) and further take into account the temperature dependence of *α*, *C* and *κ*. For *T* < *Θ*_*D*_ where *Θ*_*D*_ ~ 250 K is the few-layer MoS_2_ Debye temperature^25^, we use $\alpha \propto {T}^{3}$, $\kappa \propto {T}^{3}$ and ${C}_{v}\propto {T}^{3}$ as the functional form of the temperature dependence of these quantities^26^. We find that ${Q}^{-1}(T)={Q}_{ph}^{-1}(T)+{Q}_{cl}^{-1}+{Q}_{sub}^{-1}(T)+{Q}_{surf}^{-1}(T)$ can accurately account for the observed temperature dependence of the energy dissipation within the high and low temperature regimes (solid blue and dashed magenta lines in Fig. 4, respectively), indicating contributions from each of these dissipation pathways. The two additional fits in Fig. 4, corresponding to the dotted violet lines, demonstrate that ${Q}^{-1}(T)={Q}_{ph}^{-1}(T)+{Q}_{surf}^{-1}(T)$ cannot alone explain the observed *Q* ^−1^(*T*) within the high and low temperature ranges and that accounting for clamping and electrostatic-related losses is necessary. However, from the fittings to the data shown in Fig. 4 (solid blue and dashed magenta lines) we do find that the relative contributions from each dissipation pathway changes between the two temperature regimes. For *T* > 110 K, we find that the largest contributions to *Q* ^−1^(*T*) are from ${Q}_{ph}^{-1}(T)$ and ${Q}_{sub}^{-1}(T)$, while for *T* < 110 K the largest contributions to *Q* ^−1^(*T*) are from ${Q}_{cl}^{-1}$. Physically, this corresponds to a transition to clamping related losses for *T* < 110 K as the MoS_2_ phonon and carrier density populations are reduced with decreasing temperature. We also find negligible contributions from ${Q}_{surf}^{-1}(T)$ within both the high and low temperature regimes, which indicates that losses due to physisorbed species, for example, are negligible. Additionally, prior works on InAs nanowires^27^ have demonstrated that energy dissipation due to clamping related losses results in a total measured dissipation on the order of 10^−4^ at low temperatures, which is consistent with our results that *Q* ^−1^(*T*) ~ ${Q}_{cl}^{-1}$ ~ 10^−4^ at *T* = 4.4 K (additional detailed information regarding the data analysis can be found in the Supporting Information). The results for few-layer MoS_2_ NEMS are strikingly different than the theoretical predictions for the dominant loss mechanisms in single layer graphene^21^ (which predict ${Q}_{sub}^{-1}(T)$ to dominate) and semiconductor resonators with micron-scale thickness^28^ (which show that thermoelastic loss dominates).

It has been demonstrated previously that thermoelastic damping (dissipation stemming from the transduction of elastic energy into thermal energy via a temperature gradient established between compressed and expanded regions of the resonator) is the dominant source of dissipation in semiconductor microscale resonators^28^ with 1.5 μm thickness. According to the Zener theory for thermoelastic damping, the dissipation is expressed as

$${Q}_{Ze}^{-1}(T)=\frac{E{\alpha }^{2}T}{{C}_{p}}\frac{\omega {\tau }_{th}}{1+{\omega }^{2}{{\tau }_{th}}^{2}}$$

where *α* is the thermal expansion coefficient, *C*_*p*_ is the constant stress heat capacity, and ${\tau }_{th}={t}^{2}{C}_{p}/{\pi }^{2}\kappa$ is the thermal relaxation time. For our samples, 1/*τ*_*th*_ ~ 9 × 10^11^ Hz, which is 4 orders or magnitude larger than the fundamental frequency associated with our resonator. Within the thermoelastic model, the dissipation highly depends upon excitation frequency with ${Q}_{Ze}^{-1}(T) \sim 0$ when the product *ωτ*_*th*_ is outside of the range^14^ 0.01 ≤ *ωτ*_*th*_ ≤ 100. In our samples, *ωτ*_*th*_ ~ 2.3 × 10^−5^, which suggests that thermoelastic dissipation is not a dominant dissipation pathway within these few-layer systems.

### Long-term durability

TMDs are seen as promising 2D candidates in an effort to replace or augment current semiconducting technologies utilizing Si, Ge, and other III-V compounds. Practical applications rely on the ability to develop device architectures with long-term durability and stability. It is known that the electrical performance of multilayer MoS_2_ transistors is unstable under ambient conditions, and that the adsorption of oxygen and/or water from the environment can substantially impact the electronic properties^29^. The same holds true for chemically modified graphene transistors^30^. In the case of phosphorene, degradation of the material due to photoassisted oxidation has been shown to occur on the timescale of several hours, which markedly hinders its technological applicability^31^. In terms of TMDs-based NEMS, however, we find that a few-layer MoS_2_ resonator is capable of operating over the duration of years without loss of force sensitivity and functionality, which affords significant promise in the applicability of such mechanically-active architectures.

**Figure 5:**
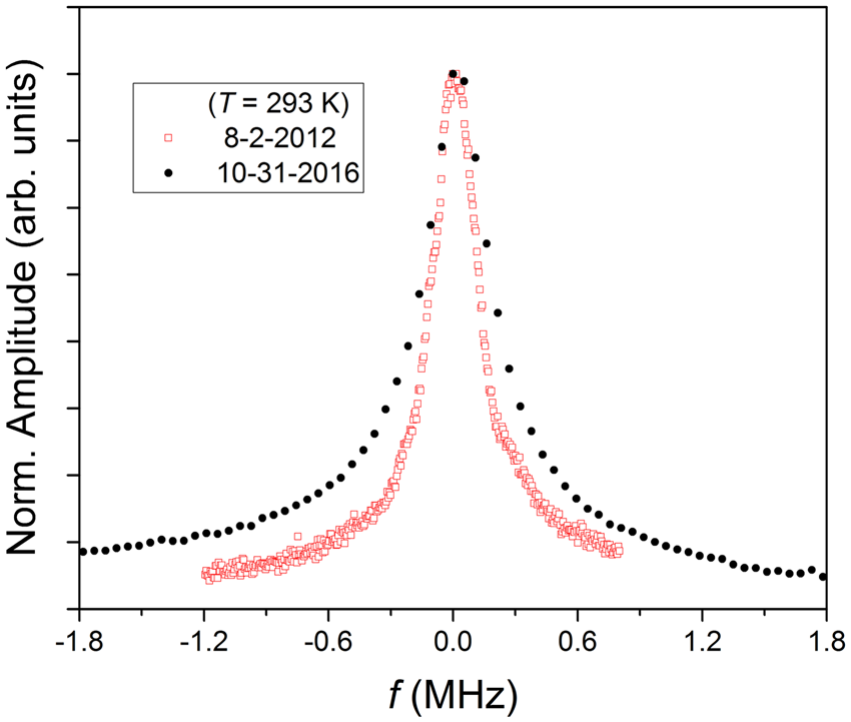
Time dependence of the fundamental mode. Normalized amplitude as a function of *f*-*f*_*O*_, where *f*_*O*_ is the fundamental frequency, measured at room temperature and at two separate times: after initial exfoliation (8-2-2012) and nearly 4 years and 3 months later (10-31-2016).

Figure 5 shows the normalized measured mechanical amplitude versus frequency at room temperature taken more than 4 years apart (data represented by the solid black circles is from the same data set shown in Fig. 2). After the initial exfoliation and measurements (8–2–2012 in Fig. 5), the sample was placed in a nitrogen-purged dry box at <20% humidity level. The quality factor decreased from ~96 to ~82 over the course of the 4 years and 3 months with only small changes in the product *Qω*, which decreased from ~1.5 × 10^10^ to ~1.1 × 10^10^. The limit on the resonator force sensitivity is given by

$$dF={(4{\kappa }_{eff}{k}_{B}T/Q\omega )}^{1/2},$$

where ${\kappa }_{eff}={m}_{eff}{\omega }^{2}=\pi {r}^{2}t\rho {\omega }^{2}$ is the effective spring constant and *k*_*B*_ is Boltzmann’s constant. We calculate the force sensitivity change from 8 fN/Hz^1/2^ to 8.2 fN/Hz^1/2^ between the time-dependent data sets shown in Fig. 5 indicating that the force sensitivity of the resonator remained effectively constant over the duration of more than 4 years.

In summary, we have measured the temperature dependence of the energy dissipation for few-layer MoS_2_ NEMS. Our results indicate that both intrinsic (phonon-phonon interactions) and extrinsic (clamping and electrostatic coupling to the substrate) dissipation sources contribute to the overall observed energy dissipation. We find that for *T* > 110 K the largest contributions to the overall dissipation are related to phonon-phonon and electrostatic interactions, while for *T* < 110 K the largest contributions to the dissipation are related to clamping losses. These results are markedly different from single-layer NEMS and micron-scale semiconductor systems, and results in a new regime due to the few-layer nature of the MoS_2_ NEMS. We further demonstrate the mechanical durability and nearly constant force sensitivity of the MoS_2_ NEMS over the time span of more than 4 years. Our results are important for understanding the fundamental limitations of few-layer NEMS at room temperature, and for the long-term applications in force and mass sensing and opto-mechanical/electro-mechanical transducers derived from few-layer materials.

## Methods

### Device Fabrication

Few-layer MoS_2_ flakes were mechanically exfoliated from bulk MoS_2_ crystals onto a SiO_2_/Si substrate with predefined circular trenches etched out of the SiO_2_. Prior to the exfoliation, standard optical lithography and reactive ion etching were used to define the trenches in the SiO_2_. No subsequent patterning or annealing was carried out following the exfoliation. A Bruker Dimension FastScan AFM was used to determine the thickness of the exfoliated flakes.

### Optical setup

The frequency spectrum of the blue diode laser, which is used for resonator excitation, is controlled with a spectrum analyzer. The reflected light intensity of the red laser, which is monitored to detect the resonator motion, is focused onto a low-noise photoreceiver after being passed through a narrow-band filter where blue wavelengths are blocked from reaching the photoreceiver. The output of the photoreceiver is measured with the spectrum analyzer thus allowing for a lock-in technique to detect the resonator motion. The cryostat is manipulated using an x-y-z stage, which allows for 250 nm precision. Laser power is recorded using a Newport Corporation power meter with a calibrated photodiode sensor.

### Time-dependent measurements

We point out that, with the exception of the data from 8-2-2012 presented in Fig. 5, all other data sets within this article were taken on or after 10-31-2016.

### Data availability

The datasets generated during and/or analyzed during the current study are available from the corresponding author on reasonable request.

## Electronic supplementary material

Supporting Information
