# Supplementary material for: Energy Dissipation Pathways in Few-Layer MoS2 Nanoelectromechanical Systems
Source: Sci Rep. 2017 Jul 18;7:5656. doi: 10.1038/s41598-017-05730-1 (PMC5515974; doi:10.1038/s41598-017-05730-1)
Supplement: Supplementary file 1 — Supporting Information [file 41598_2017_5730_MOESM1_ESM.pdf]

## Supporting Information

# Energy Dissipation Pathways in Few-Layer $MoS_2$ Nanoelectromechanical Systems

Bernard R. Matis[1, *], Brian H. Houston[1], and Jeffrey W. Baldwin[1]

[1]Naval Research Laboratory, Code 7130, Washington, DC 20375, United States

*Correspondence to: bernard.matis@nrl.navy.mil

### Influence of chamber pressure on mechanical response:

Exposure of the $MoS_2$ resonator to a gas has a notable impact on the measured mechanical response. In Fig. S1 we plot the measured mechanical amplitude as a function of frequency for two separate cryostat chamber pressures: under vacuum and at atmospheric pressure. With exposure to atmosphere, the frequency of the fundamental mode increases by ~0.9 MHz while the full-width-at-half-maximum, $\Delta f$, increased by ~1.3 MHz. The presence of the gas within the chamber significantly dampens the resonator motion such that the quality factor, $Q$, decreases from > 90 to ~16, and the product $Q\omega$ = Q*($2\pi f$) decreased by an order of magnitude. All experimental results presented within the main text were carried out with the cryostat chamber under vacuum (pressure ~ $10^{-6}$ Torr), and after an extended pump down period

(on the order of several days minimum) to assist in the removal of physisorbed water on the surface of the resonator and to ensure minimal energy dissipation due to gas friction.

An estimate of the dissipation due to gas friction[S1] at room temperature and under vacuum can be made using the expression $Q_{gas}^{-1}(T) = \frac{P}{\pi \rho v_{avg}}\left(1 + {}^{t}/_{d}\right)\sqrt{{}^{8\mu}/_{\pi RT}}$ where $P \sim 1.0 \times 10^{-6}$ Torr is the gas pressure, $\rho \sim 5{,}060$ kg/m$^3$ is the $MoS_2$ resonator density, $v_{avg} \sim 500$ m/s is the mean speed of thermal-molecular motion based upon the equipartition theorem, $t$ and $d$ are the resonator thickness and diameter, respectively, $\mu \sim 0.028966$ kg/mol is the mean molecular weight of the gas, and $R = 8.314$ J/Kmol is the universal gas constant. We assume the same heat capacities at constant volume and constant pressure. The result is $Q_{gas}^{-1}(T) \sim 9 \times 10^{-14}$ at $T = 293$ K, which is 9 orders of magnitude lower than the lowest measured $Q^{-1}$ values reported in the main text.

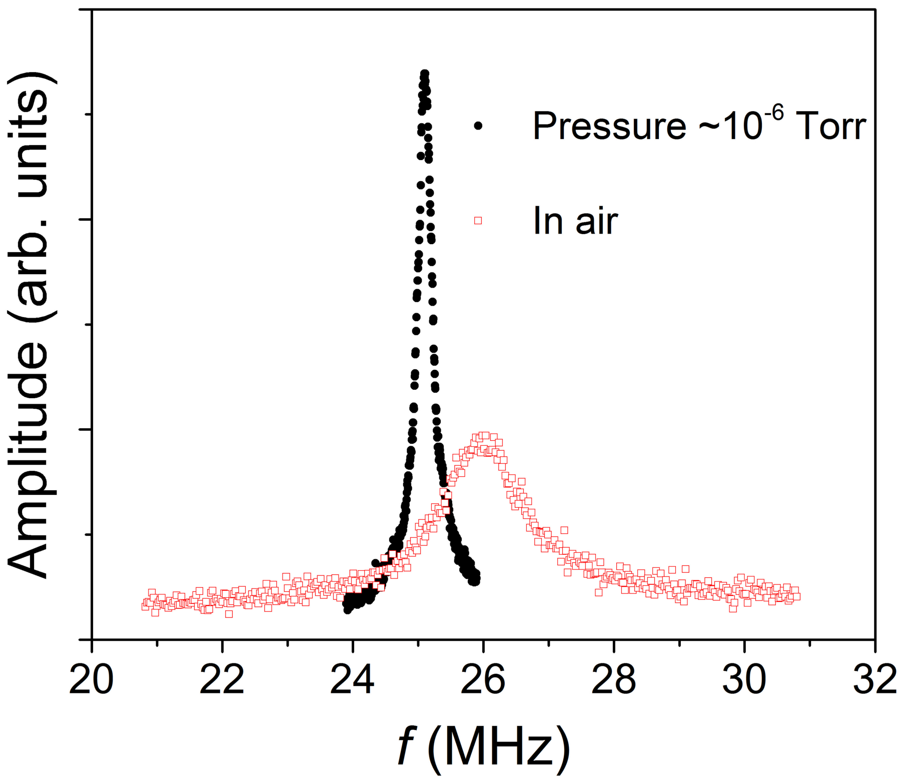


**Figure S1 | Influence of gas friction on mechanical response.** Amplitude as a function of frequency, *f*, for the 17-nm-thick $MoS_2$ resonator showing the fundamental mode for measurements with the cryostat at atmospheric pressure (red, open squares) and under vacuum (solid black circles). Data was taken during the initial measurements after the resonator fabrication (8-2-2012 data set in Fig. 5 of the main text).

**Cubic fit to *f*(*T*):**

The cubit fit to the data shown in Fig.3d of the main text captures the functional form of the fundamental frequency temperature dependence, and is used in the subsequent data analysis of $Q^{-1}(T)$. We find that $f(T)$ is well fitted by the following cubic equation:

$$f(T) = 0.34891T^3 - 219.87198T^2 + 4771.34682T + 3.0466x10^7$$

The fit to this functional form for $f(T)$, shown in Fig. 3d of the main text, had an $R^2$ value of ~0.99975.

**Akheiser regime discussion:**

Dissipation due to phonon-phonon interactions is modelled using Akheiser theory (see page 9 of the main text) across the full range of temperatures (4.4 K – 293 K). The Akheiser theory is applicable in our resonators since $1/\tau_{ph} >> \omega$ where $\tau_{ph}$ is the phonon relaxation time (~ $2x10^{-12}$ s) and ω the resonator angular frequency (~$1x10^8$ $s^{-1}$). Temperature dependent Raman measurements on few-layer $MoS_2$ samples have shown a slight decrease in the full-width at half-maximum (FHWM) of the $A_{1g}$ mode with decreasing temperature from 523 K to 83 K[S2]; the temperature-dependent linewidth of the Raman peak can be used to estimate phonon relaxation times using the Heisenberg uncertainty relation $FWHM \sim \Delta\varepsilon = \hbar/\tau_{ph}$ where $\hbar$ is Planck's constant divided by 2π. The data shown in Fig. 4 of Reference S2 yields $\tau_{ph}$ ~ 1.6 ps at 300 K and $\tau_{ph}$ ~ 1.97 ps at 83 K, which demonstrates that $\tau_{ph}$ changes by a factor of only ~ 1.2 between

room temperature and 83 K. The temperature-dependent Raman data from Reference S2 also show an apparent saturation in the FWHM for $T < 200$ K. These results demonstrate that the order of magnitude of $\tau_{ph}$ (on the order of $1.0\text{x}10^{-12}$) remains the same from room temperature down to temperatures below 100 K, which ensures that $1/\tau_{ph} >> \omega$ and that the Akheiser regime remains applicable in few-layer $MoS_2$ devices at low temperatures.

**Temperature-dependent data analysis:**

The data fittings shown in Fig. 4 of the main text were carried out by using the expression $Q^{-1}(T) = \sum A_n Q_n^{-1}(T)$ where $A_n$represents a constant coefficient and free-fitting parameter and $Q_n^{-1}(T)$ is a dissipation pathway. The figure 4 data fittings were found by fitting the high and low temperature data (above and below 110 K) to $Q^{-1}(T) = R * Q_{ph}^{-1}(T) + L + (M * T) + S * Q_{surf}^{-1}(T)$, where $R$, $L$, $M$ and $S$ are constants and are used as the free-fitting parameters; $Q_{ph}^{-1}(T)$ and $Q_{surf}^{-1}(T)$ are determined using the known expressions provided in the main text, $L$ represents the contribution from temperature-independent clamping losses ($Q_{cl}^{-1}$), and the term $M*T$ corresponds to the dissipation from electrostatic interactions between the charges in the resonator and the doped silicon substrate ($Q_{sub}^{-1}(T)$). We choose $R$ and $S$ as the free fitting parameters to $Q_{ph}^{-1}(T)$ and $Q_{surf}^{-1}(T)$ for several reasons. First, the quantities appearing within the expressions for $Q_{ph}^{-1}(T)$ and $Q_{surf}^{-1}(T)$, specifically $C_v$, $C_p$, $\gamma$, $\rho$, $v$, $E$ and $\alpha$ have all been experimentally determined for $MoS_2$ and we use these experimentally determined quantities within the data fittings ($E$ is the Young's modulus found for our $MoS_2$ resonator, which is reported on page 7 of our manuscript). Second, the fitting parameters $R$ and $S$ contain additional information such as mode-participation factors[S3] and information related to device geometry and

structure[S4]. Prior studies[S3,S4] have accounted for such factors and information by incorporating them into a constant of proportionality term to each dissipation pathway, which is the convention that we adopt for our data analysis. Third, the scattering times $\tau_{ph}$ ~ 2 ps and $\tau_s$ ~ 1 μs (the scattering times associated with $Q_{ph}^{-1}(T)$ and $Q_{surf}^{-1}(T)$, respectively) are separated by six orders of magnitude and it is this large difference between the scattering times that helps to separate the relative contributions of $Q_{ph}^{-1}(T)$ and $Q_{surf}^{-1}(T)$ to the overall measured dissipation, which is why we refrain from using the scattering times as free fitting parameters in the data fittings.

The following Table S1 summarizes the results of the data fittings, and the dissipation contributions from each pathway (right four columns of Table S1) can be compared to the overall measured dissipation, $Q^{-1}(T)$, presented in Fig. 4 of the manuscript ($Q^{-1}(T)$ ~ $10^{-2}$ at $T$ = 293 K and $Q^{-1}(T)$ ~ $10^{-4}$ at $T$ = 4.4 K):

**Table S1 | Data analysis summary.** Results of the data fittings shown in Fig. 4 of the main text, which includes the free fitting parameters *R*, *M* and *S*, and the contributions from the four dissipation pathways. Within the chart, the values reported in the last four columns are found by multiplying *R*, *M* and *S* by the known expressions for $Q_{ph}^{-1}(T)$, $Q_{sub}^{-1}(T)$ and $Q_{surf}^{-1}(T)$ that are described within the main text. The dominant dissipation pathways have been highlighted in red (for $T > 110$ K) and purple (for $T < 110$ K).

| R | M | S | $R{\times}Q_{ph}^{-1}(T)$ | $Q_{cl}^{-1}$ (=$L$) | $M{\times}Q_{sub}^{-1}(T)$ | $S{\times}Q_{surf}^{-1}(T)$ |
|---|---|---|---|---|---|---|
| 118,667 ($T > 110$ K) | $1.1 \times 10^{-5}$ ($T > 110$ K) | $4 \times 10^{-14}$ ($T > 110$ K) | $10^{-2}$ ($T = 293$ K) | $10^{-18}$ ($T > 110$ K) | $10^{-3}$ ($T = 293$ K) | $10^{-20}$ ($T = 293$ K) |
| $1.1 \times 10^{-7}$ ($T < 110$ K) | $1 \times 10^{-6}$ ($T < 110$ K) | 12,542 ($T < 110$ K) | $10^{-21}$ ($T = 4.4$ K) | $10^{-4}$ ($T < 110$ K) | $10^{-6}$ ($T = 4.4$ K) | $10^{-10}$ ($T = 4.4$ K) |

**References:**


S1: Braginsky, V. B., Mitrofanov, V. P. & Panov, V. I. *Systems with Small Dissipation*. The University of Chicago Press: Chicago, IL, pp 20-21, (1985).

S2: Sahoo, S., Gaur, A. P. S., Ahmadi, M., Guinel, M. J.-F. & Katiyar, R. S. Temperature-Dependent Raman Studies and Thermal Conductivity of Few-Layer $MoS_2$. *J. Phys. Chem. C.* **117,** 9042-9047 (2013).

S3: Metcalf, T. H., Pate, B. B., Photiadis, D. M. & Houston, B. H. Thermoelastic damping in micromechanical resonators. *Appl. Phys. Lett.* **95,** 061903 (2009).

S4: Lee, J., Wang, Z., He, K., Shan, J. & Feng, P. X.-L. Air damping of atomically thin $MoS_2$ nanomechanical resonators. *Appl. Phys. Lett.* **105,** 023104 (2014).
